# Worsening pulmonary outcomes during sex reassignment therapy in a transgender female with cystic fibrosis (CF) and asthma/allergic bronchopulmonary aspergillosis: a case report

**DOI:** 10.1186/s12890-020-01272-x

**Published:** 2020-08-31

**Authors:** G. Y. Lam, J. Goodwin, P. Wilcox, B. S. Quon

**Affiliations:** 1 Centre for Heart Lung Innovation, St. Paul’s Hospital, University of British Columbia, 1081 Burrard Street, Vancouver, BC V6Z 1Y6 Canada; 2 Adult Cystic Fibrosis Program, St. Paul’s Hospital, Vancouver, BC Canada

**Keywords:** Cystic fibrosis, Asthma, ABPA, FEV1, Estrogen, Progesterone

## Abstract

**Background:**

Cystic Fibrosis (CF) is a hereditary pulmonary and extra-pulmonary disease that occurs equally in men and women. However, a difference in morbidity and mortality rates between the sexes has been long documented. Similarly, a sex-disparity in disease severity has been reported in asthma as well. Studies done to date point to estrogen as a possible cause of this sex disparity in pulmonary outcomes in both conditions.

**Case presentation:**

Here, we describe a case of a patient with CF and asthma/allergic bronchopulmonary aspergillosis (ABPA) undergoing sex reassignment therapy (male-to-female) and the negative impact it had on her lung function and frequency of pulmonary exacerbations in the context of increasing doses of exogenous estrogen.

**Conclusions:**

This case raises the possibility of a link between estrogen and worsening pulmonary outcomes and the need for further studies into transgender individuals with CF and/or asthma/ABPA as well as those undergoing high dose estrogen therapy for other indications.

## Background

Cystic Fibrosis (CF) is an autosomal recessive genetic disorder that commonly results in chronic lung disease, occurring with equal frequency in both men and women. Unfortunately, registry data from around the world has long documented the existence of a “Gender Gap”, wherein female CF patients have significantly higher morbidity and mortality rates than males [1]. Specifically, women have not only been found to have a shortened life expectancy when compared to their male counterparts but also experience more pulmonary exacerbations, faster lung function decline, lower BMI and higher rates of CF related complications, such as CF-related diabetes [1]. Given that girls and boys with CF have similar health outcomes prior to puberty, and that lung function is lower and pulmonary exacerbation rates are higher during phases of the menstrual cycle associated with higher levels of estrogen, estrogen is thought to be a major mediator of this sex disparity [2].

A similar gap in outcomes has been reported in allergic and inflammatory airway conditions whereby women with asthma tend to experience more frequent and severe exacerbations compared to men [3]. However, the effect of estrogen on asthma is more controversial. The risk of asthma was noted to increase in post-menopausal women taking estrogen replacement therapy (ERT) compared to those who do not use ERT [4, 5]. On the other hand, other studies have either failed to demonstrate any significant changes in forced expiratory volume in 1 sec (FEV_1_) with ERT [6] while another group even documented improvements in lung function and asthma exacerbation rates post estrogen therapy [7]. Thus, the effect of exogenous estrogen on asthma and other allergic pulmonary conditions remains controversial.

A number of researchers have proposed possible mechanisms for estrogen-mediated worsening of CF airways disease including increased IL-8 and T-helper 17 (Th17) responses leading to increased neutrophilic inflammation, depletion of airway surface liquid via modulation of mucosal ion transport, increased mucus production and viscosity [1], promotion of *Pseudomonas aeruginosa* growth, and the selection of bacterial virulence factors that enhance the protective mucoid phenotype [8]. Here, we present a unique case report of a patient receiving exogenous estrogen treatment as part of sex reassignment therapy and discuss its potential adverse impact on her pulmonary and atopic outcomes.

## Case presentation

A 23-year-old transgender female (male-to-female) patient with CF (mutations ΔF508/394delTT) and baseline percent-predicted forced expiratory volume in 1 s (ppFEV_1_) of 60–65% (2.5–2.6 L) has been followed by the adult CF clinic since 2015. She has chronic sputum growth of *Pseudomonas aeruginosa*, *Stenotrophomonas maltophilia*, methicillin sensitive *Staphylococcus aureus*, *Aspergillus fumigatus* and *Scedosporium apiospermum*. Her comorbid conditions include eczema, chronic rhinosinusitis with nasal polyposis, pancreatic insufficiency, low bone mineral density, CF dysglycemia, nephrolithiasis, and depression/anxiety.

Her medical history is also significant for asthma and allergic bronchopulmonary aspergillosis (ABPA) requiring treatment with voriconazole, inhaled amphotericin B and prolonged courses of prednisone prior to transitioning to the adult clinic. She has been maintained on montelukast 10 mg daily, Symbicort 6/200mcg 2 puffs daily and Pulmicort 200mcg 2 puffs daily. She was reinitiated on anti-fungal therapy with itraconazole for presumed ABPA exacerbation in January 2018 due to a sudden spike in IgE level accompanied by worsening pulmonary symptoms, including wheeze and chest tightness. She was subsequently started on benralizumab in August 2019 due to a persistently elevated serum eosinophil count with good biochemical response.

**Fig. 1:**
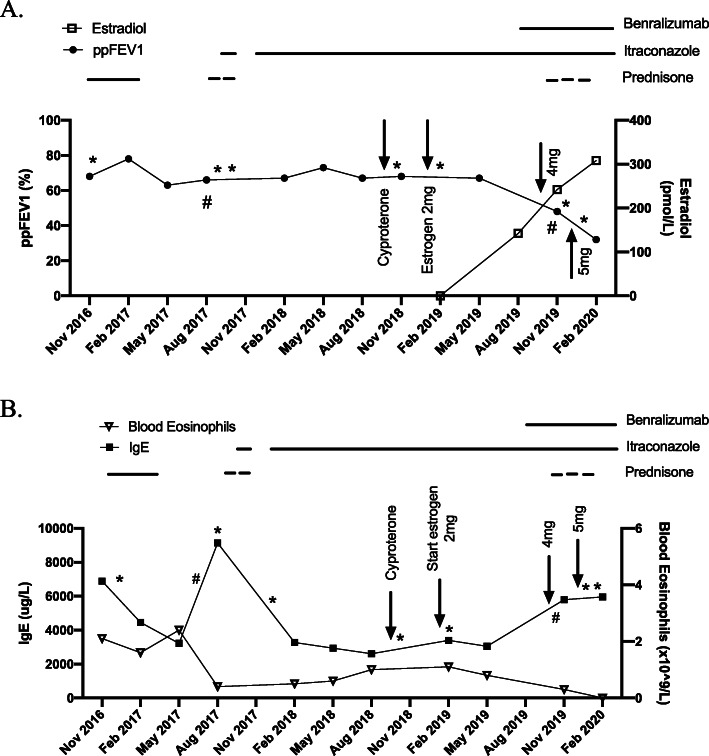
**a** Lung Function and Estradiol Change Over Time. Relative stability in ppFEV_1_ (left sided Y axis; solid circles) until the introduction and escalation of exogenous estrogen therapy with rising serum estradiol levels (right sided Y axis; open squares), in spite of ongoing itraconazole, pulse steroid and benralizumab treatment (period of treatment denoted by the horizontal bars). Arrows denote start and dose adjustment dates of cyproterene and estrogen. * denotes pulmonary exacerbations requiring oral antibiotic treatment. # denotes pulmonary exacerbations requiring intravenous antibiotic treatment. Percent predicted forced expiratory volume in 1 s (ppFEV_1_) was determined in accordance with Global Lung Function Initiative 2012 spirometry equations. **b** Blood Eosinophil and IgE Change Over Time. Sustained rise in IgE levels (left sided Y axis; solid squares) corresponding to estrogen therapy in spite of ongoing itraconazole, pulse steroid and benralizumab treatment (period of treatment denoted by the horizontal bars). Serum eosinophil levels (right sided Y axis; open triangles) drop in response to benralizumab therapy. Arrows denote start and dose adjustment dates of cyproterene and estrogen. * denotes pulmonary exacerbations requiring oral antibiotic treatment. # denotes pulmonary exacerbations requiring intravenous antibiotic treatment

Despite her comorbidities and frequent pulmonary exacerbations (PEx; 2–3 per year), she had relatively stable lung function (Fig. 1a). However, over the last 6 months, she experienced a precipitous decline in ppFEV_1_ and a further increase in the frequency of PEx. Of note, she began sex reassignment therapy with estrogen at 2 mg daily dose starting February 2, 2019, followed by a dose increase to 4 mg daily on Sept 19, 2019 and to 5 mg daily on December 2, 2019 (Fig. 1a). She has been on cyproterone (anti-androgen therapy) at 25 mg daily without any overt clinical side effects since October 27, 2018. Within 2–4 weeks of estrogen start and with each dose increase, she experienced a PEx, requiring treatment with oral or IV antibiotics and prednisone but without recovery to previous baseline following symptomatic improvement from most episodes. Repeated sputum cultures failed to identify any new infectious organisms throughout this time. Concurrently, she also experienced a steady increase in her total IgE level (Fig. 1b) and worsening eczema despite treatment with itraconazole 300 mg total daily dose, pulses of prednisone 50 mg daily with taper, and benralizumab. No other biologics have been trialed as the patient failed to access omalizumab due to medication coverage issues and dupilumab has not yet received approval for use in Canada for severe asthma.

## Discussion and conclusions

The sex disparity in clinical outcomes between female and male individuals with CF and other allergic or inflammatory diseases is a concerning and poorly understood phenomenon. Here, we present a unique case of a patient with relatively stable CF lung disease complicated by both asthma and ABPA who then experienced a rapid decline in lung function and increased frequency of PEx temporally corresponding to rising serum levels of estradiol as part of ongoing sex reassignment therapy. Given the rise in the number of individuals now identifying as transgender (doubling from 700,000 to 1.4 million over the last decade) in the US [9], this case highlights the need for physicians to recognize the potential impact that estrogen might have on pulmonary outcomes in CF. As such, patients who wish to undergo sex reassignment therapy should be counseled about the possibility of worsening pulmonary and/or allergic outcomes and closely monitored for potential clinical deterioration during therapy. While we cannot definitively conclude that estrogen therapy is the cause of her lung function decline, the persistent loss of lung function despite aggressive treatment with oral and intravenous antibiotics and the temporal relationship between estrogen dose escalation and the onset of PEx implicates estrogen as a plausible contributor. This case therefore highlights the need for clinicians to counsel their patients on the possibility of worsening pulmonary outcomes prior to their patient initiating estrogen therapy and the need for close monitoring post-initiation. Ultimately, the decision to discontinue estrogen therapy or not following any observed clinical deterioration depends on patient preference based on a discussion of the ongoing risks and benefits; in our case, the patient elected to continue treatment despite the drop in lung function.

The patient described in this case has the added complexity of co-existing asthma and ABPA. In vitro and in vivo studies in CF and asthma have demonstrated that estrogen, in either condition, is linked to a more profound pro-inflammatory response during periods of PEx [1, 3]. Since the clinical presentation of CF lung disease and asthma/ABPA can be quite similar, it is difficult to conclude which of these diseases has been exacerbated by exogenous estrogen use. The rising IgE level certainly suggests that her asthma/ABPA is at least playing a role in her decline. However, given that she is on comprehensive ABPA and asthma therapy with evidence of response previously (drop in IgE level with prednisone and itraconazole therapy in September 2017) and currently (drop in serum eosinophil count since starting benralizumab; Fig. 1b), we believe her allergic conditions cannot fully explain her worsening respiratory status. The deteriorating clinical outcome in response to estrogen might be mediated by both her CF and asthma/ABPA. This case therefore highlights the potential risk of estrogen therapy in CF patients and especially in those with comorbid allergic conditions.

Estrogen, as well as its agonists and antagonists, are not only prescribed in context of sex reassignment therapy. For example, as CF patients are getting older, more women will be entering menopause and considering hormone replacement therapy. Currently, there is a lack of literature on the clinical outcomes in post-menopausal CF patients with or without estrogen replacement therapy. The doses used in hormone replacement and sex reassignment therapy are often higher than in oral contraceptive pills. Therefore, further studies of post-menopausal and transgender female CF patients will be needed to fully appreciate the possible harms of high dose estrogen use in these small but growing populations.

## Data Availability

Data sharing is not applicable to this article as no datasets were generated or analysed during the current study.

## Abbreviations

CF: Cystic Fibrosis
PEx: Pulmonary exacerbation
ABPA: Allergic Bronchopulmonary Aspergillosis
FEV1: Forced Expiratory Volume in 1 s

## References

[1] Sweezey NB, Ratjen F (2014). The cystic fibrosis gender gap: potential roles of estrogen. Pediatr Pulmonol.

[2] Raghavan D, Jain R (2016). Increasing awareness of sex differences in airway diseases. Respirology.

[3] Bonds RS, Midoro-Horiuti T (2013). Estrogen effects in allergy and asthma. Curr Opin Allergy Clin Immunol.

[4] Romieu I, Fabre A, Fournier A, Kauffmann F, Varraso R, Mesrine S, Leynaert B, Clavel-Chapelon F (2010). Postmenopausal hormone therapy and asthma onset in the E3N cohort. Thorax.

[5] Dratva J (2010). Use of oestrogen only hormone replacement therapy associated with increased risk of asthma onset in postmenopausal women. Evid Based Med.

[6] Hepburn MJ, Dooley DP, Morris MJ (2024). The effects of estrogen replacement therapy on airway function in postmenopausal, asthmatic women. Arch Intern Med.

[7] Haggerty CL, Ness RB, Kelsey S, Waterer GW (2003). The impact of estrogen and progesterone on asthma. Ann Allergy Asthma Immunol.

[8] Chotirmall SH, Smith SG, Gunaratnam C, Cosgrove S, Dimitrov BD, O'Neill SJ, Harvey BJ, Greene CM, McElvaney NG (2012). Effect of estrogen on pseudomonas mucoidy and exacerbations in cystic fibrosis. N Engl J Med.

[9] Flores AR, Herman JL, Gates GJ, Brown TNT (2016). How many Adults Identify as Transgender in the United States?.

